# Italian physiotherapists’ knowledge of and adherence to osteoarthritis clinical practice guidelines: a cross-sectional study

**DOI:** 10.1186/s12891-021-04250-4

**Published:** 2021-04-23

**Authors:** Simone Battista, Stefano Salvioli, Serena Millotti, Marco Testa, Andrea Dell’Isola

**Affiliations:** 1 Department of Neurosciences, Rehabilitation, Ophthalmology, Genetics, Maternal and Child Health, University of Genova, Campus of Savona, Genova, Italy; 2 Clinical Epidemiology Unit, Orthopedics, Department of Clinical Sciences Lund, Lund University, Lund, Sweden

**Keywords:** Osteoarthritis, Osteoarthritis, knee, Osteoarthritis, hip, Practice guidelines as topic, Clinical governance, Physical therapy specialty, Physical therapists, Education, public health professional

## Abstract

**Introduction:**

Implementation of clinical practice guidelines (CPGs) to manage musculoskeletal conditions among physiotherapists appears suboptimal. Osteoarthritis is one of the most disabling conditions worldwide and several studies showed a lack of knowledge of and adherence to osteoarthritis CPGs in physiotherapists’ clinical practice. However, those studies are not conclusive, as they examine the knowledge of and adherence to CPGs only in isolation, or only by focussing on a single treatment. Thus, analysis of the knowledge of and adherence to CPGs in the same sample would allow for a better understanding of the evidence-to-practice gap, which, if unaddressed, can lead to suboptimal care for these patients. This study aims at assessing Italian physiotherapists’ evidence-to-practice gap in osteoarthritis CPGs.

**Methods:**

An online survey divided into two sections investigating knowledge of and adherence to CPGs was developed based on three high-quality, recent and relevant CPGs. In the first section, participants had to express their agreement with 24 CPG statements through a 1 (completely disagree) to 5 (completely agree) scale. We defined a ≥ 70% agreement with a statement as consensus. In the second section, participants were shown a clinical case, with different interventions to choose from. Participants were classified as ‘Delivering’ (all recommended interventions selected), ‘Partially Delivering’ (some recommended interventions missing) and ‘Non-Delivering’ (at least one non-recommended interventions selected) the recommended intervention, depending on chosen interventions.

**Results:**

822 physiotherapists (mean age (SD): 35.8 (13.3); female 47%) completed the survey between June and July 2020. In the first section, consensus was achieved for 13/24 statements. In the second section, 25% of the participants were classified as ‘Delivering’, 22% as ‘Partially Delivering’ and 53% as ‘Non-Delivering’.

**Conclusions:**

Our findings revealed an adequate level of knowledge of osteoarthritis CPGs regarding the importance of exercise and education. However, an adequate level of adherence has yet to be reached, since many physiotherapists did not advise weight reduction, but rest from physical activity, and often included secondary treatments (e.g. manual therapy) supported by low-level evidence. These results identify an evidence-to-practice gap, which may lead to non-evidence based practice behaviours for the management of patients with osteoarthritis.

## Introduction

Osteoarthritis is the most prevalent joint disease, and one of the most common causes of disability worldwide [1], for whose management several international Clinical Practice Guidelines (CPGs) have been released [2–4]. CPGs are collections of graded recommendations supported by a systematic review of evidence, intended to help clinicians to optimise patient care [5]. In osteoarthritis, the European League Against Rheumatism (EULAR), Osteoarthritis Research Society International (OARSI) and the National Institute for Health and Care Excellence (NICE) CPGs [2–4] recommend exercise and education [6] as first-line interventions for their ability to reduce pain and disability, regardless of the severity of the disease [7, 8].

Despite the differences in grading the strength of recommendations between them, all abovementioned CPGs categorised the level of evidence, of the different treatments, into six categories from Ia to IV [2–4]. Treatments categorised as Ia are derived from systematic reviews of randomised controlled trials (RCTs) [2–4, 9]; whereas, treatments categorised from IIa to IV are based on lower quality RCTs, cohort studies or the opinion of experts in the field [2–4, 9]. The level of evidence in all CPGs is one of the factors that contribute to defining the strength of the recommendations together with the balance between benefits and harms, considerations of values and preferences, and resources implications [10]. By taking into account all these factors, treatments such as therapeutic exercise and patients education (Ia, level of evidence) are categorised as first-line interventions, whereas treatments such as hyaluronic acid injection and manual therapy (IV, level of evidence) are categorised as conditional recommendations [2–4].

In spite of the availability of several high-quality osteoarthritis CPGs, Egerton et al. highlighted that clinicians who work with patients with osteoarthritis perceived themselves as under-prepared and unfamiliar with CPGs [11]. Besides, implementation of CPGs for the management of musculoskeletal conditions among physiotherapists appears suboptimal [12] and osteoarthritis often remains under-diagnosed and under-treated, with less than 40% of people with osteoarthritis receiving the recommended first-line intervention [13, 14].

Several studies have explored physiotherapists’ knowledge of and adherence to osteoarthritis CPGs, showing major gaps in the implementation of weight reduction strategies, therapeutic exercise and patients education, as opposed to what has been noticed for other passive modalities (e.g. manual therapy) [15–19]. The reasons behind this gap are several. First of all, the care of people with knee and hip osteoarthritis is a complex process in which the clinician is required to balance knowledge of the best evidence with the patients’ preferences and beliefs [20]. Secondly, clinicians may face several barriers to the implementation of CPGs, such as clinical applicability, language and lack of time, which can widen this gap even further [21–23].

However, the aforementioned studies on the application of osteoarthritis CPGs are not conclusive, as they either examined the knowledge of or adherence to CPGs, [16–19] or focus solely on a particular treatment (e.g. therapeutic exercise) [15]. In fact, knowledge of the CPGs does not automatically translate into clinical practice [24, 25]*,* thus an analysis of the knowledge of and adherence to CPGs in the same sample would allow for a better understanding of the so-called*evidence-to-practice gap* [21].

In line with this, this study aimed at exploring the knowledge of and adherence to osteoarthritis CPGs in a cohort of Italian physiotherapists in order to identify the possible evidence-to-practice gap*.* By analysing this gap in Italy, this study gathered information that might be more easily transferred to other Mediterranean countries which seem to have higher educational needs compared to the Northern-European ones [26].

## Methods

### Study design

A quantitative web-basedcross-sectional survey investigating physiotherapists’ knowledge of and adherence to osteoarthritis CPGs was developed according to the *International Handbook of Survey Methodology* and to the *Checklist for Reporting Results of Internet E-Survey* through the use of distinct and iterative steps [27, 28]. The study was conducted following the Declaration of Helsinki. Ethical approval was obtained from the Ethics Committee for University Research (CERA: Comitato Etico per la Ricerca di Ateneo), University of Genova (approval date: 15/06/2020; CERA2020.07) and follows the Strengthening the Reporting of Observational Studies in Epidemiology (STROBE) recommendations for reporting observational studies [29].

### Survey development

The questionnaire was developed based on the EULAR, OARSI and NICE CPGs [2–4]. Before the online dissemination, the survey was tested on a sample of six physiotherapists specialised in musculoskeletal rehabilitation. The online version of the questionnaire was delivered through *Microsoft 365 Forms*, a secure web application to build and manage online surveys and databases, respecting the European General Data Protection Regulations [30]. The questionnaire included a brief cover letter, and the informed consent outlining the aim of the study. The cover letter emphasised that participation in the survey was voluntary and that anonymity and confidentiality were guaranteed.

The questionnaire was delivered in Italian and it was divided into two sections investigating (1) the knowledge of and (2) the adherence to osteoarthritis CPGs. Before the first section, a paragraph investigating the socio-demographic variables (e.g. sex, age, years of experience) and if the physiotherapists read, at least, one osteoarthritis CPG, was included. The first section comprised 24 statements on knee and hip osteoarthritis management, adapted from the aforementioned CPGs. Each statement was acquired from the synoptic review of the three CPGs (Table 1).

**Table 1:** Section 1: Statements and synoptic review of Clinical Practice Guidelines

Statements	Clinical Practice Guidelines
1) Exercise can be effective on all patients, regardless of the pain severity.	NICE (1.2.5–1.4.1) [4]; EULAR (3–6-7) [2]; OARSI (Tables 2-3) [3]
2) In an advanced stage of the disease, exercise can damage the joint (**reversed statement**).	NICE (1.2.5–1.4.1) [4]; EULAR (2–3–6-7) [2]; OARSI (Tables 2-3) [3]
3) The rehabilitation programme must always include a part of education on the pathophysiology of osteoarthritis and self-management strategies.	NICE (1.3.1–1.3.2–1.3.3) [4]; EULAR (3–5) [2]; OARSI (Tables 2-3) [3]
4) The rehabilitation programme should always include a part of manual treatment (**reversed statement**)	NICE (1.4.2) [4]; EULAR (−); OARSI (−)
5) Exercise should only be undertaken after prescribing drug treatment to control pain (**reversed statement**).	NICE (1.2.5–1.4.1) [4]; EULAR (3–6-7) [2]; OARSI (Tables 2-3) [3]
6) The use of topical anti-inflammatory drugs is effective for pain relief for knee osteoarthritis.	NICE (1.5.3) [4]; EULAR (−); OARSI (Table 2) [3]
7) Radiographic findings are needed to express a functional diagnosis of osteoarthritis (**reversed statement**).	NICE (1.1.1) [4]; EULAR (1) [2]; OARSI (−)
8) Radiographic findings are needed to plan the physiotherapy treatment (**reversed statement**).	NICE (1.1.1) [4]; EULAR (1) [2]; OARSI (−)
9) Physical activity should be avoided because it can damage the joint (**reversed statement).**	NICE (1.2.5–1.4.1) [4]; EULAR (3–6-7) [2]; OARSI (Tables 2-3) [3]
10) The use of topical anti-inflammatory drugs is effective for pain relief for hip osteoarthritis.	NICE (−); EULAR (−); OARSI (−)
11) In case of severe joint degeneration, it is necessary to recommend rest from physical activity (**reversed statement**).	NICE (1.2.5–1.4.1) [4]; EULAR (2–3–6-7) [2]; OARSI (Tables 2-3) [3]
12) In cases of severe pain (VAS ≥ 6/10), arthroplasty surgery should be preferred to rehabilitation (**reversed statement**).	NICE (1.6) [4]; EULAR (−); OARSI (−)
13) The use of TENS should be considered.	NICE (1.4.4) [4]; EULAR (−); OARSI (−)
14) The use of physical therapies such as lasers, TECAR and ultrasound therapy should be considered (**reversed statement**).	NICE (1.4.4) [4]; EULAR (−); OARSI (−)
15) In addition to the rehabilitation treatment, it is useful to recommend physical activity (for example, yoga, swimming, Nordic walking).	NICE (1.2.5–1.3.2–1.4.1) [4]; EULAR (−); OARSI (Tables 2-3) [3]
16) It is important to recommend weight loss to overweight or obese patients.	NICE (1.2.5–1.4.3) [4]; EULAR (3–8) [2]; OARSI (Tables 2-3) [3]
17) Age > 45, pain and absence of joint stiffness (or < 30 min) in the morning are sufficient to diagnose osteoarthritis.	NICE (1.1.1) [4]; EULAR (−); OARSI (−)
18) The use of comfortable footwear, braces or aids should be considered.	NICE (1.3.2–1.4.7–1.4.8–1.4.9) [4]; EULAR (3–9-10) [2]; OARSI (Tables 2-3) [3]
19) It is advisable to refer the patient for arthroscopy surgery to reduce symptoms and start/continue treatment (**reversed statement**).	NICE (1.4.10) [4]; EULAR (−); OARSI (−)
20) It is necessary to assess the impact of osteoarthritis on function, quality of life and disability.	NICE (1.2.1) [4]; EULAR (1) [2]; OARSI (−)
21) At least 10–12 sessions are needed to ensure proper treatment for osteoarthritis.	NICE (1.4.1) [4]; EULAR (6) [2]; OARSI (−)
22) In the treatment for osteoarthritis, the patient’s adherence to the treatment must be motivated.	NICE (1.3.2–1.4.1–1.7.1) [4]; EULAR (−); OARSI (−)
23) Joint hyaluronic acid and/or corticosteroid infiltrations should be considered.	NICE (1.5.12–1.5.13) [4]; EULAR (−); OARSI (Table 2) [3]
24) The supplements of chondroitin and glucosamine should be considered (**reversed statement).**	NICE (1.4.5) [4]; EULAR (−); OARSI (−)

Legend: (n), CPGs paragraph into which the statements were originally reported; (−), the CPGs did not adopt a position on that statement; [n], CPGs reference

If disagreement was found between the CPGs, the most recent recommendation was considered when phrasing the statement. To measure agreement with the statement, we used a 5-point Likert scale ranging from completely disagree (score 1) to completely agree (score 5) [31]. Participants who partially or completely agreed (scores 4–5) were considered to agree with the statements. Furthermore, to limit acquiescence bias, i.e. the tendency to agree with all the survey statements, eleven reversed statements were put into the questionnaire so that disagreement with those statements (scores 1–2) would indicate an agreement with the CPGs [32].

The second section presented a clinical vignette illustrating a case of knee osteoarthritis (Table 2). Clinical vignettes are considered a valid tool to assess healthcare professionals’ clinical reasoning and behaviour, including physiotherapists, as they are easy to administer, and all the variables within them can be easily manipulated [33].

**Table 2:** Section 2: Clinical vignette proposed treatments

Clinical Scenario: Maria, a 72-year-old housewife, lives with her husband, who is in good health. She cultivates the hobby of gardening. For the past ten years, she has been suffering from knee pain which, in certain periods, forces her to take NSAIDs and to limit daily activities for a few days. Over the past two years, the pain has become increasingly frequent (VAS 5/10), so that she has decided to find some help with the housework and she is struggling to take care of the garden. She also suffers from diabetes and is overweight (BMI 28). She decides to consult her physician, who recommends her to do a visit to the physiotherapist.		
**Management:**		
Core Treatment	Partially Core Treatment	Non-Core Treatment
• Evaluation and planning of the rehabilitation treatment; • Weight loss advice.	• Referral to the physician for drug therapy.	• Referral to the physician for arthroscopic surgery (joint debridement); • Referral to the physician for prosthetic intervention.
**Assessment:**		
Core Treatment		
• Assessment of the quantity and quality of pain; • Assessment of the function; • Assessment of disability and participation.		
**Treatment:**		
Core Treatment	Partially Core Treatment	Non-Core Treatment
• Specific exercise on the joint (muscle strengthening); • Generic exercise (aerobic exercise or generic physical activity); • Education on the pathophysiology of osteoarthritis.	• Manual therapy (mobilisation and/or massage); • TENS; • Load reduction devices (braces, insoles or walking aids); Hyaluronic acid and corticosteroid injections.	• Activity rest (reduce the load on the joint); • Other physical therapies (Laser, Ultrasound etc.); • Supplement integration: glucosamine and chondroitin
For how many sessions would you treat this patient? • For less than 5 sessions; • Between 5 and 10 sessions; • For more than 10 sessions.		

The participants were, therefore, invited to express how they would manage that specific patient by selecting from a list of options. The options were grouped into three sub-sections representing three different clinical moments (management, assessment and treatment). In the management section, the participants were asked whether they would opt to treat the presented patient or refer her to a specialist for pharmacological or surgical treatment. Participants who decided to refer the patient to a specialist without performing any assessment or treatment were directed to the end of the questionnaire, considered non-delivering any possible clinical options.

### Participants

An online version of the questionnaire, attainable through a hyperlink, was delivered through the Italian Association of Physiotherapists (AIFI) and the University of Genova newsletters. We included physiotherapists who had treated at least one person with osteoarthritis in the previous six months within the targeted population. To do so, after the cover letter, the questionnaire included a preliminary question asking the respondents if they had treated any patients, with hip or knee osteoarthritis in the last six months. Participants who answered “No” were shown a Thank-You page and were not allowed to continue the questionnaire.

### Variables

The primary outcome of the present study was the level of knowledge of and adherence to CPGs of a sample of Italian physiotherapists.

### Analysis

Descriptive analysis was carried out to underRicerca di Ateneo), University ofstand the sample’s characteristics. Moreover, the frequencies of physiotherapists who declared to have read at least one osteoarthritis CPG was reported.

#### Section 1: level of knowledge of clinical practice guidelines – statement consensus

In section one, participants who partially or completely agreed with a statement (scores 4–5) or partially or completely disagreed (scores 1–2) with a reversed statement were considered to agree with the CPGs recommendation. The overall consensus with each statement was investigated. In the absence of a standard threshold, we defined a ≥ 70% agreement with a statement as consensus [34].

#### Section 2: level of adherence to clinical practice guidelines – clinical vignette

In section two, participants were classified as ‘Delivering’, ‘Partially Delivering’ and ‘Non-Delivering’ the core treatments depending on the interventions chosen. Briefly, they were considered as delivering the recommended intervention if they chose all the treatments recommended by the CPGs for the patient described in the vignette, without selecting non-recommended treatments. They were considered partially delivering the recommended intervention if they chose only some of the recommended treatments but none of the non-recommended ones. Lastly, they were considered as non-delivering the recommended intervention if they chose at least one of the non-recommended treatments or if they decided either not to treat the patient or to treat her for fewer than five sessions, and therefore the percentage of physiotherapists’ Delivering’, ‘Partially Delivering’ and ‘Non-Delivering’ the recommended intervention was calculated.

## Results

### Participants

**Fig. 1:**
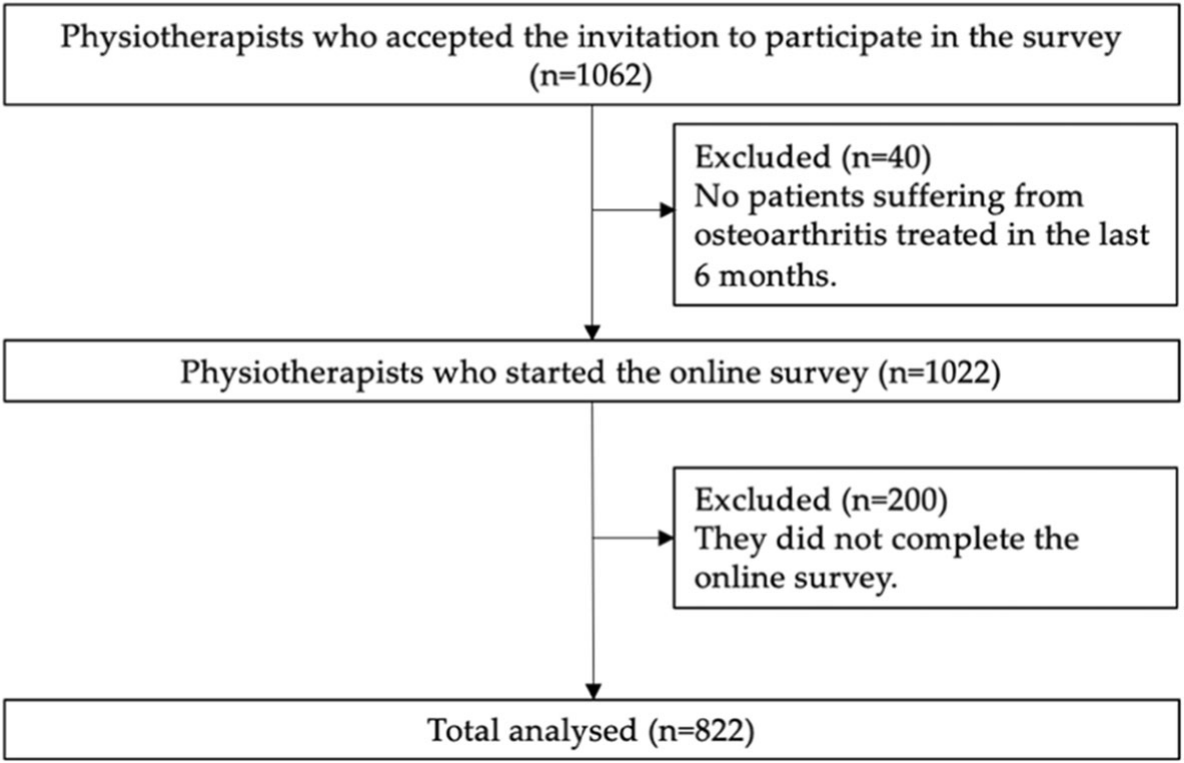
Participants’ Flowchart

Through the AIFI and the University of Genova newsletter, we were able to reach a total of 1582 physiotherapists, of which 1062 (response rate: 67%) completed the online survey between 16 June 2020 and 6 July 2020. Among them, 40 (4%) had not treated any patient with osteoarthritis in the previous six months, and 200 (19%) did not complete the survey in all its sections. Thus, 822 (77%; (mean age (SD):35.8 (13.3); female 47%; male 53%) physiotherapists compiled the questionnaire in all its sections (Fig. 1) and were included in the analysis (Table 3). Of these, 465 physiotherapists (57%) declared to have read at least one osteoarthritis CPG, whereas 357 (43%) did not.

**Table 3:** Participants’ demographic characteristics

Demographic Characteristics	
**Age (years)(mean,(SD))**	35.77 (13.3)
**Sex** (female); (male) (N (%)):	387 (47); 435 (53)
**Years of Practice** (N (%)):	
Less than 1 year	87 (11)
From 1 to 5 years	319 (39)
From 6 to 10 years	149 (18)
More than 10 years	267 (32)
**Highest Academic Level Reached** (N (%)):	
Bachelor of Science (BSc)/Equivalent title	282 (34)
Post-Graduate I Level Degree*	382 (47)
Master of Science (MSc)/Post-Graduate II Level Degree†	122 (15)
Doctor of Philosophy (PhD)	36 (4)
**Read at least one osteoarthritis CPGs** (N (%)):	
Yes	465 (57)
No	357 (43)

Legend: N, number; %, percentage; *Academic degree that can be gained after BSc (Italian education system); † Academic degree that can be gained after MSc (Italian education system)

#### Section 1: level of knowledge of clinical practice guidelines – statement consensus

**Fig. 2:**
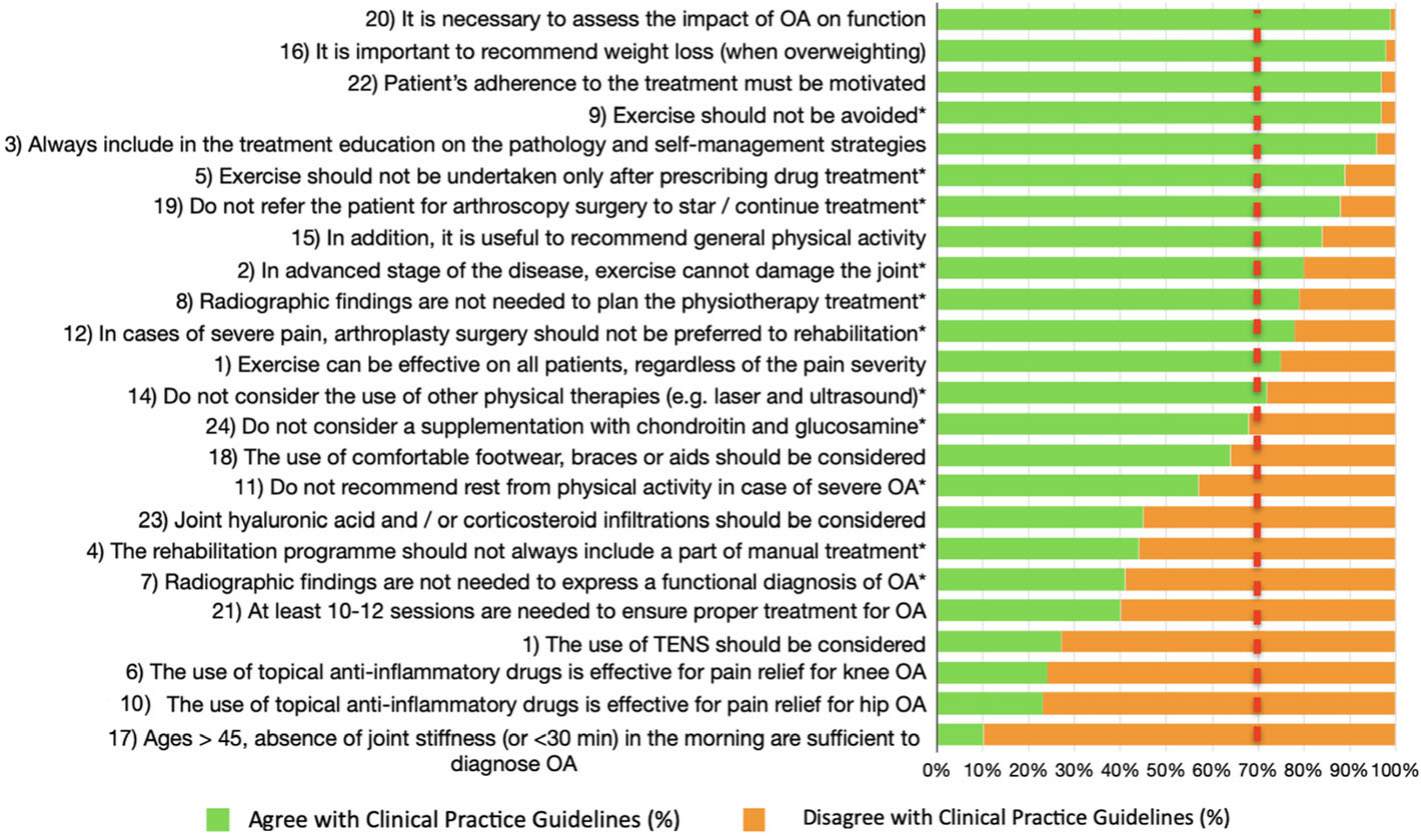
Level of agreement with Osteoarthritis (OA) Clinical Practice Guidelines Statements. * Statement originally reversed in the questionnaire. † The red line represents the consensus threshold set at 70%. ‡ The statements are reported in a shortened version, see Table 1 for reference

Overall, consensus was achieved for 13 (54%) statements (1–3, 5, 8, 9, 12, 14–16, 19, 20, 22) out of 24 (Fig. 2).

These statements addressed the role of clinical assessment, exercise, education, weight loss, and the effectiveness of physical therapies in the management of people with knee or hip osteoarthritis. Conversely, the consensus was not reached for those statements dealing with the role of supplements, radiographic findings, manual therapy, topical non-steroidal drugs, TENS, the number of sessions and the criteria for clinical diagnosis.

#### Section 2: level of adherence to clinical practice guidelines – clinical vignette

Demographic characteristics, percentages of the selection of each item and the classification of the participants in ‘Delivering’, ‘Partially Delivering’ and ‘Non-Delivering’ the recommended intervention are reported in Table 4 and Table 5.

**Table 4:** Participants’ profile by level of adherence for the Clinical Vignette

	‘Delivering’ (N = 202)	‘Partially Delivering’ (N = 181)	‘Non-Delivering’ (N = 439)
**Age** (years)(mean,(SD))	31.2 (10.9)	37.4 (13.7)	37.2 (13.7)
**Sex** (N (%)):			
Female	81 (40)	86 (48)	220 (50)
Male	121 (60)	95 (52)	219 (50)
**Years of Practice** (N (%)):			
Less than 1 year	30 (15)	18 (10)	39 (9)
From 1 to 5 years	100 (50)	71 (39)	148 (34)
From 6 to 10 years	40 (20)	28 (16)	81 (18)
More than 10 years	32 (15)	64 (35)	171 (39)
**Highest Academic Level Reached** (N (%)):			
Bachelor of Science (BSc)/Equivalent level	57 (28)	64 (35)	161 (37)
I Level Master Degree*	115 (57)	79 (44)	188 (43)
Master of Science (MSc)/II Level Master†	23 (11)	32 (18)	67 (15)
Doctor of Philosophy (PhD)	7 (4)	6 (3)	23 (5)
**Read at least one osteoarthritis CPGs** (N (%)):			
Yes	124 (61)	114 (63)	240 (55)
No	78 (39)	67 (37)	199 (45)

Legend: N, number; %, percentage; *Academic degree that can be gained after BSc (Italian education system); † Academic degree that can be gained after MSc (Italian education system)

**Table 5:** Frequencies of answers to Clinical Vignette by level of adherence with Clinical Practice Guidelines

Question	All (*N* = 822; 100%)		‘Delivering’ (N = 202; 25%)		‘Partial Delivering’ (N = 181; 22%)		‘Non-Delivering’ (N = 439; 53%)	
	YES	NO	YES	NO	YES	NO	YES	NO
**Section 1: Management** (N (%))								
1) Physiotherapy treatment	794 (97)	28 (3)	202 (100)	0 (0)	181 (100)	0 (0)	411 (94)	28 (6)
2) Referral pharmacological	159 (19)	663 (81)	40 (20)	162 (80)	15 (8)	166 (92)	104 (24)	335 (76)
3) Referral for debridment	15 (2)	807 (98)	0 (0)	202 (100)	0 (0)	181 (100)	15 (3)	424 (97)
4) Referral for surgery	26 (3)	796 (97)	0 (0)	202 (100)	0 (0)	181 (100)	26 (6)	413 (94)
5) Weight Loss	645 (79)	177 (21)	202 (100)	0 (0)	90 (50)	91 (50)	353 (80)	86 (20)
**Section 2: Assessment** (N (%))								
1) Pain	727 (88)	95 (12)	202 (100)	0 (0)	138 (76)	43 (24)	387 (88)	52 (12)
2) Functionality	756 (92)	66 (8)	202 (100)	0 (0)	164 (91)	17 (9)	390 (89)	49 (11)
3) Disability and Partecipation	722 (88)	100 (12)	202 (100)	0 (0)	149 (82)	32 (18)	371 (85)	68 (15)
**Section 3: Treatment** (N (%))								
1) Load reduction (rest)	244 (30)	578 (70)	0 (0)	202 (100)	0 (0)	181 (100)	244 (56)	195 (44)
2) Manual therapy	612 (75)	210 (25)	145 (72)	57 (28)	135 (75)	46 (25)	332 (76)	107 (24)
3) Muscles strengthening	702 (85)	120 (15)	202 (100)	0 (0)	143 (79)	38 (21)	357 (81)	82 (19)
4) Generic exercise (e.g. aerobic exercise or generic physical activity)	525 (64)	297 (36.)	202 (100)	0 (0)	92 (51)	89 (49)	231 (53)	208 (47)
5) Education on the pathophysiology of osteoarthritis	698 (85)	124 (15)	202 (100)	0 (0)	142 (78)	39 (21)	354 (81)	85 (19)
6) TENS	111 (14)	711 (86)	25 (12)	177 (88)	27 (15)	154 (85)	59 (13)	380 (87)
7) Other Physical Therapies (e.g. ultrasound and laser)	137 (17)	685 (83)	0 (0)	202 (100)	0 (0)	181 (100)	137 (31)	302 (69)
8) Load reduction devices (e.g. braces, insoles or walking aids).	185 (23)	637 (77)	30 (15)	172 (85)	34 (19)	147 (82)	121 (28)	318 (72)
9) Hyaluronic acid and corticosteroids	111 (14)	711 (86)	7 (4)	195 (96)	19 (11)	162 (89)	85 (19)	354 (81)
10) Treatment: Supplements	279 (34)	543 (66)	0 (0)	202 (100)	0 (0)	181 (100)	77 (18)	362 (82)
**Section 4: Number of sessions** (N (%))*								
Less than 5 sessions	71 (9)	0 (0)	0 (0)	71 (17)	Less than 5 sessions	71 (9)	0 (0)	0 (0)
Between 5 and 10 sessions	465 (58)	138 (68)	121 (67)	206 (50)	Between 5 and 10 sessions	465 (58)	138 (68)	121 (67)
More than 10 sessions	258 (33)	64 (32)	60 (33)	134 (33)	More than 10 sessions	258 (33)	64 (32)	60 (33)

Legend: N, number; %, percentage; *Percentage calculated on *N* = 794; *N* = 28 could not access to this section as they didn’t check the “Management section: 1) Physiotherapy treatment” option

The ‘Delivering’ group (*N* = 202; 25%) provided the patient with all the CPGs recommended treatments. In the ‘Partially Delivering’ group (*N* = 181; 22%), all the participants performed the physiotherapy treatment, but only half delivered weight loss advice. The majority of the sample assessed functionality, disability, participation, pain and, delivered muscle-strengthening exercises and education. General exercise (e.g. aerobic exercise and general physical activity) was prescribed by about half of the group. Finally, in the ‘Non-Delivering Group’ (*N* = 439; 53%), the majority of participants performed the physiotherapy treatment. As far as non-recommended treatments are concerned, load reduction (rest), and other physical therapies (e.g. ultrasound and laser) were the most often delivered. Among the recommended treatments, the ones that were delivered by the majority of the members of the group were muscle strengthening and pathophysiology education. About half of this group prescribed generic exercises (aerobic exercise or generic physical activity).

## Discussions

Clinical practice guidelines are an important tool that aims at bridging the gap between best evidence and clinical practice. Although our study highlighted an overall good level of knowledge of the core first-line intervention, adherence to CPGs was low. Many physiotherapists did not advise losing weight, but advised rest, while often including secondary treatments (e.g. manual therapy) supported by low-level evidence.

Most physiotherapists (> 90%) participating in the survey were aware of the importance of therapeutic exercise, education and enhancing patients’ adherence to the treatment, in the caretaking process of people with hip or knee osteoarthritis. These results are in line with the ones reached by other physiotherapists worldwide [16–19]. Despite this, 56% of the physiotherapists participating in the survey considered it essential to include manual therapy (e.g. manual mobilisation, massage) in the treatment. Current evidence shows that when manual therapy is compared with exercise therapy alone, it provides only short term benefits in reducing pain, improving function, and physical performance [35]. However, this conclusion on manual therapy is gathered from low-quality evidence, and therefore CPGs rated it as lower quality when compared to the evidence supporting exercise, which makes manual therapy only a conditional treatment [3, 4]. In line with this, prioritising manual therapy in patients’ management may reduce the time allocated to exercise. However, we did not ask the participants whether they considered manual therapy more effective than exercise, or which treatment they would prioritise, thus leaving uncertainties regarding the clinical impact of this finding. Future studies, with a mixed-method design are needed to better understand how different treatments are weighed by clinicians in the management of people with osteoarthritis.

Furthermore, we found an insufficient level of knowledge in three distinct areas: a) the criteria for the clinical diagnosis of osteoarthritis, b) the role of other non-surgical interventions that could enhance therapeutic exercise benefits (e.g. topical anti-inflammatory drugs and TENS), 3) the number of sessions needed to ensure an optimal outcome. As far as the clinical diagnosis and drug prescription are concerned, this lack of knowledge might be due to the fact that, in Italy, physiotherapists are not allowed to perform clinical diagnosis and prescribe drugs. However, they are often the first health care professionals that people with osteoarthritis refer to. Thus, they should be aware of the recommended pharmacological management to facilitate the integration of drug therapy with physiotherapy and the proper clinical diagnostic criteria in order to refer patients to relevant healthcare professionals, when necessary.

Our study showed that only 10% of the respondents considered being 45 years old or older, having pain, and joint stiffness for less than 30 min in the morning, sufficient criteria for the diagnosis of osteoarthritis, as recommended by the NICE CPGs. These seemed to be the most appropriate criteria to ensure that even younger patients with osteoarthritis woul receive appropriate care in line with CPGs [36]. The lack of agreement regarding clinical diagnostic criteria may partially explain the relatively high percentage (> 40%) of physiotherapists who considered radiographic findings necessary to express a clinical diagnosis of osteoarthritis. This data is consistent with the findings of a similar survey conducted by Ayanniyi et al. [19]. Radiographic findings should be taken into account only when other diseases are the suspected cause of the symptoms (e.g. infection, cancer, rheumatoid arthritis) or when surgical intervention is planned [4]. These recommendations are based on evidence that shows a weak association between the severity of radiographic findings and pain and disability levels [37, 38]. Furthermore, basing clinical decisions on imaging fosters the perception of osteoarthritis as a wear-and-tear disease which may, in turn, induce fear-avoidance behaviours [38, 39]. However, since physiotherapists in Italy are unable to prescribe radiographic investigations, the impact of this finding on the clinical management of the patients is uncertain.

The second section of the survey showed mismatching between the knowledge of CPGs and their application in clinical practice. Although most physiotherapists showed adequate knowledge of first-line interventions, only a minority (25%) wholly adhered to CPGs, whereas more than 50% included at least one non-recommended strategy or treatment.

One of the challenges in the implementation of CPGs seems to be the fact that health care professionals view osteoarthritis as a “non-serious” disease. This may depend on inadequate preparation at undergraduate level [11, 20, 40]. These erroneous beliefs might be carried on in post-graduate degrees, since the percentage of “Non-Delivering” did not change throughout the different levels of academic degrees achieved (> 50%). Moreover, in Italy, there are no MSc degrees available, specifically on musculoskeletal and rheumatic conditions, and PhD curricula are very specific, therefore, if not focussed on osteoarthritis, they cannot bridge the pre-existingevidence-to-practice gap. In addition, implementing osteoarthritis CPGs in the complex setting of clinical care can be challenging [20] since clinicians have to face several barriers among which patients’ preferences, resource availability, discrepancies between CPGs, lack of English knowledge and limited access to information [22].

Although the patient in the clinical vignette was overweight and presented with moderate symptoms, the interviewed physiotherapists often excluded advising weight loss, whilst rest was often considered. In fact, recommending weight loss may be considered by some physiotherapists as beyond their clinical scope [41]. This data is in line with other studies showing that both Australian and British physiotherapists recommended muscle strengthening exercises, but seemed less confident in prescribing aerobic exercise and recommending weight loss [18, 41]. Providing physiotherapists with additional specific training aimed at dealing with overweight patients may enhance the patients’ outcomes and increase the overall level of adherence to CPGs.

The interpretation of the relatively high inclusion of rest as well as the load reduction in the treatment is difficult to explain, especially in light of the good level of knowledge shown in the first part of the questionnaire. However, the CPGs available do not specify how to adapt the therapeutic exercise in those cases with severe osteoarthritis symptoms, where pain can be easily triggered by joint movement or weight-bearing activities. This is also highlighted by the fact that about 50% of the physiotherapists in the “Non-Delivering” group declared to have read at least one osteoarthritis CPG. Thus, in light of these results, it can be hypothesised that physiotherapists may feel unsure and unprepared when having to deal with this pain condition [42]. Discrepancies between CPGs knowledge and application may also depend on factors that are external to the physiotherapist, and they may vary by country. Regarding osteoarthritis CPGs, this is the first study that pointed towards this discrepancy, starting from several CPGs and by considering a plethora of treatments.

Beneath the differences between the three groups, a transversal trait was found regarding the application of manual therapy which was delivered by more than 70% of the sample. From a cultural perspective, manual therapy is a core competence of physiotherapy, which set the basis of this professional figure in the past, and patients often expect this type of treatments from physiotherapists [40]. Meeting patients’ expectations is thought to foster a positive clinician-patient relationship while enhancing the treatment outcome by inducing analgesia, regulating patients’ emotions, and reorganising the body’s mental representations [43, 44]. Therefore, this data may reflect the contrast between treatments recommendations and patients’ expectations, which can be itself the results of a specific cultural belief that needs to be investigated.

Some limitations of this study need to be discussed. Firstly, our sample was mainly based on physiotherapists who completed a post-graduate degree, therefore our results might overestimate the real level of knowledge of and adherence to osteoarthritis CPGs. Secondly, we did not investigate the participants’ clinical practice setting (e.g. private practice, public care etc.) which might have had an impact on the participants’ level of adherence to CPGs.

Our findings revealed that Italian physiotherapists are aware of the core treatments for patients with osteoarthritis. However, they showed a low level of knowledge of the clinical diagnostic criteria and of the usefulness of other non-surgical treatments that can support first-line intervention (e.g. TENS and non-steroidalanti-inflammatory medications). Moreover, an adequate level of adherence is yet to be reached. These results identify an evidence-to-practice gap which may lead to non evidence-based practice behaviours for the management of the patients with hip and knee osteoarthritis.

Finding new strategies to bridge the gap between evidence and clinical practice appears to be necessary, therefore providing physiotherapists with CPGs in their native language and fostering their use through university programmes could be one of the possible solutions proposed. Moreover, the use of recognised manuals aimed at developing CPGs is advocated. These should ascertain that all search stages are documented for transparency and reproducibility and that the most important elements for a real practical implementation, such as algorithms for clinical decision-making for complicated cases, and patients’ inclusion-exclusion criteria, are included [45].

Finally, the professional image of physiotherapists within society should be reconceptualised. In particular, we should continue to foster a new vision of physiotherapists, as no longer anchored to treatments that are mainly based on physical and manual therapy, but as figures whose treatment paradigm focusses on improving the patient’s individual functioning by specific treatment strategies, such as exercise and education, that take into account scientific evidence conveyed into specific contexts.

## Data Availability

The datasets used and analysed during the current study are available from the corresponding author on reasonable request.
